# Osteoradionecrosis of the mandible: a case series at a single institution

**DOI:** 10.1186/1916-0216-42-46

**Published:** 2013-09-11

**Authors:** Artur Gevorgyan, Kevin Wong, Ian Poon, Nick Blanas, Danny J Enepekides, Kevin M Higgins

**Affiliations:** 1 Department of Otolaryngology - Head and Neck Surgery, Sunnybrook Health Sciences Centre, 2075 Bayview Avenue, Room M1-102, Toronto, ON, M4N 3M5, Canada; 2 Department of Radiation Oncology, Sunnybrook Health Sciences Centre, 2075 Bayview Avenue, Room M1-102, Toronto, ON, M4N 3M5, Canada; 3 Department of Dentistry, Sunnybrook Health Sciences Centre and Odette Cancer Centre, University of Toronto, Toronto, ON, Canada

**Keywords:** Osteoradionecrosis, Radiotherapy, Head and neck neoplasms, Mandible, Mandibulectomy, Hyperbaric oxygenation, Free tissue flaps

## Abstract

**Background:**

Osteoradionecrosis (ORN) defines exposed irradiated bone, which fails to heal over a period of 3–6 months without evidence of residual or recurrent tumor. In the previous decades, a staging and treatment protocol suggested by Marx, has dominated the approach to ORN. However, recently this paradigm is shifting. The purpose of this study was to evaluate our institutional experience in managing ORN through a retrospective review of case series from a large urban academic cancer centre.

**Methods:**

A retrospective chart review was conducted to include all ORN cases from 2003 to 2009 diagnosed at the Department of Otolaryngology – Head and Neck Surgery and the Department of Dentistry. The staging of ORN was assessed as affected by tumor site, tumor stage, radiotherapy modality and dose, chemotherapy, dental work, and time to diagnosis. The effectiveness of hyperbaric oxygen therapy (HBO) and surgery in the management of ORN was evaluated.

**Results:**

Fourteen cases of ORN were documented (incidence 0.84%). Primary subsites included tonsils, tongue, retromolar trigone, parotid gland, soft palate and buccal mucosa. There were 5 (35.7%) stage 1, 3 (21.4%) stage 2, and 6 (42.9%) stage 3 cases. ORN severity was not significantly associated with gender, smoking, alcohol use, tumor site, T stage, N stage, AJCC stage, or treatment modality (radiation alone, surgery with adjuvant radiation or adjuvant chemoradiation). Patients treated with intensity-modulated radiotherapy developed less severe ORN compared to those treated with conventional radiotherapy (p < 0.015). ORN stage did not correlate with radiation dose. In one patient only dental procedures were performed following radiation and could be implicated as the cause of ORN. HBO therapy failed to prevent ORN progression. Surgical treatment was required for most stage 2 (partial resections and free tissue transfers) and stage 3 patients (mandibulectomies and free tissue transfers, including two flaps in one patient). At an average follow up of 26 months, all patients were cancer-free, and there was no evidence of ORN in 84% of patients.

**Conclusions:**

In early ORN, we advocate a conservative approach with local care, while reserving radical resections with robust reconstruction with vascularized free tissue for advanced stages.

## Background

Osteoradionecrosis (ORN) is defined as exposed irradiated bone that fails to heal over a period of 3 months without a residual or recurrent tumor [1]. Some of the signs and symptoms include pain, drainage, fistulization to mucosa or skin, trismus, malocclusion, swelling, and food impaction. There is an ongoing argument about the exact definition of ORN, and specifically about timing, radiographic and histologic features determining the diagnosis [2].

In 1983, Marx proposed a theory of pathogenesis of ORN, in conjunction with a staging system and a management algorithm [3]. He implicated the role of radiation-induced hypocellularity, hypovascularity, and hypoxia as leading factors in the development of ORN. Recently, the proposition of the fibroathropic theory does not only imply atrophy of cellular components forming the bone, but also activation of the fibroblastic lineage, with eventual replacement of tissue with abnormal myofibroblasts, resulting in ORN [4].

The clinical risk factors of ORN can be divided into local and systemic. Some of the local factors include tumor stage, tumor site, dose or radiation (>50-60 Gy), radiation field, dental extraction and poor oral hygiene, whereas systemic factors include infection, immune deficient states, co-morbidities and malnutrition.

Despite challenges to Marx’s theories, his staging system is still widely accepted. Stages 1 disease includes exposed alveolar bone without signs of pathologic fracture, which responds to hyperbaric oxygen (HBO) therapy. Stage 2 disease does not respond to HBO, and requires sequestrectomy and saucerization, whereas stage 3, which involves full thickness bone damage or pathologic fracture, usually requires complete resection and reconstruction with free tissue.

In 2004, a French multicentre randomized, double-blind, placebo-controlled trial demonstrated no benefit of HBO treatment [5]. There were no significant differences in terms of ORN recovery, time to treatment failure and time to pain relief. In none of the outcome measures did HBO outperform placebo. Interestingly, this study was stopped at the second interim analysis after showing potentially worse outcomes for HBO.

In addition, a recent review showed a lack of higher levels of evidence in ORN studies, and questioned the usefulness of HBO therapy, suggesting reserving it to early stage disease only [6] While HBO remains a part of the treatment scheme, surgical treatment has come to the forefront of the management of advanced ORN. Aggressive surgical resection of all diseased hard and soft tissue and immediate reconstruction with free tissue transfer has been suggested for stage 3 disease [7].

The rationale of the present study was to summarize the institutional experience with the management of ORN at a large urban cancer centre, emphasizing the early and aggressive surgical treatment employed to prevent the progression of ORN.

## Methods

A retrospective chart review was conducted on patients presenting with ORN to the Department of Otolaryngology – Head and Neck Surgery and the Department of Dentistry at a large urban cancer centre. This study was approved by the institutional Research Ethics Board. All head and neck cancer patient diagnosed and treated from ORN from January of 2003 until December of 2009 were included in this study. The documented patient characteristics included: gender, age, tobacco and alcohol consumption, and Karnofsky Performance Status. Clinical parameters included: primary site, TNM and AJCC disease stage, pathological diagnosis, initial treatment (surgery, radiation, chemotherapy, or a combination), time from radiation to ORN, radiation modality and dose, pre-radiation and post-radiation dental treatments, use of hyperbaric oxygen therapy, as well as frequency and type of surgical procedures used in ORN management. ORN stage was classified according to Marx’s criteria [3].

The HBO protocol involved dives of 100% oxygen for 90 minutes under either 2.4 or 2.0 atmospheres of pressure. It included 30 dives preoperatively and 10 dives postoperatively for diagnosed ORN cases. Twenty preoperative dives were used for prophylaxis prior to elective surgery on irradiated patients at risk, i.e. dental extractions in the irradiated field. HBO therapy was never used alone as a treatment modality, but as an adjunct to surgical debridement.

Follow up was documented from the clinic notes and included tumor recurrence and ORN resolution. It was available for 92.9% of patients.

Statistical analysis was performed using SPSS for Mac (Version 19.0, IBM Corp., Somers, NY). Chi-square test was used for categorical data. One-way ANOVA with Tukey’s multiple comparisons test was used for continuous data. P value of <0.05 was considered statistically significant.

## Results

### Patient characteristics

During the interval of the study (7 years) 1575 patients were treated with radiation for head and neck cancer. During the same time frame, 14 patients were treated for ORN, with the ORN incidence rate of 0.89% (Table 1). The majority of patients were males (92.4%) in their 50s (mean patient age 57.9 years). Most patients were smokers (78.6%) and drinkers (64.3%). There were no statistically significant differences in the proportions of male patients, smokers or drinkers according to ORN stage. Karnofsky Performance Status was available in 7 patients, and showed that these were mostly high functioning individuals.

**Table 1:** Patient characteristics

	**Stage 1**	**Stage 2**	**Stage 3**	**Total**	**p value**
Number of patients	5 (35.7%)	3 (21.4%)	6 (42.9%)	14	N/A
Number of males (%)	5 (100%)	2 (66.7%)	6 (100%)	13 (92.4%)	p = 0.21^∞^
Smokers (%)	4 (80%)	3 (100%)	4 (66.7%)	11 (78.6%)	p = 0.75^∞^
Alcohol use	4 (80%)	2 (66.7%)	3 (50%)	9 (64.3%)	p = 0.77^∞^
Mean age, years	–	–	–	54.45	N/A
^§^KPS (mean ± SD)	–	–	–	88.57 ± 3.78	N/A

^∞^ Freeman-Halton extension of the Fisher exact probability test.

^§^*KPS* Karnofsky Performance Status (data available in 7 patients, not analyzed).

### Tumor characteristics

Tumor characteristics and initial treatment are presented in Table 2. The majority of tumors were tonsillar (5 of 14) or tongue (4 of 14) carcinomas, with single cases of retromolar trigone, parotid gland, soft palate and buccal mucosa tumors. Pathologically all tumor were classified as squamous cell carcinomas, excluding an adenocarcinoma of the parotid gland. A minority of tumors was early stage (stage II – 35.7%), while the majority were advanced tumors (stage III, 28.6%, and stage IV, 35.7%).

**Table 2:** Primary tumor site, stage, pathology and initial treatment

**Primary site**	**Number of patients**	**TNM stage**	**AJCC stage**	**Pathology**	**Initial treatment**	**ORN Stage**
		T4N2	IVA	SCC	Surgery + adjuvant CCRT	2
		T4N2	IVA	SCC	RT	1
Tonsil	5	T4N1	IVA	SCC	RT	3
		T2N0	II	SCC	RT	3
		T2N0	II	SCC	RT	3
		T3N0	III	SCC	Surgery + adjuvant CCRT	2
Tongue	4	T3N0	III	SCC	Surgery + adjuvant RT	1
		T2N2	IVA	SCC	Surgery + adjuvant CCRT	3
		T2N1	III	SCC	Surgery + adjuvant RT	2
Retromolar trigone	2	T2N0	II	SCC	RT	1
		T2N0	II	SCC	RT	1
Parotid gland	1	T2N2	IVA	Adenocarcinoma	Surgery + adjuvant RT	3
Soft palate	1	T3N0	III	SCC	RT	1
Buccal mucosa	1	T2N0	II	SCC	RT	3

*CCRT* concomitant chemoradiotherapy, *RT* radiotherapy, *SCC* squamous cell carcinoma.

### ORN severity and modifying factors

All ORN lesions were confined to the mandible (100%). There were 35.7% stage 1, 21.4% stage 2 and 42.9% stage 3 ORN lesions. ORN severity by stage was not significantly associated with gender (p = 0.139), smoking (p = 0.514), alcohol use (p = 0.583), tumor site (p = 0.381), T stage (p < 0.429), N stage (p = 0.643), AJCC stage (p = 0.231), or treatment modality (radiation alone, surgery with adjuvant radiation or adjuvant concomitant chemoradiation, p = 0.163), (Chi-square tests).

The severity of ORN was positively associated with radiation modality (Table 3), with intensity-modulated radiotherapy (IMRT) resulting in less severe and conventional radiotherapy leading to more severe ORN cases (p < 0.015). There was no correlation identified between radiation dose and severity of ORN. ORN was diagnosed after an average of 26.85 months from the end of radiotherapy, with a trend (but no statistical significance) of stage 3 ORN being discovered later.

**Table 3:** ORN severity in relation to radiation modality, dose and time to diagnosis

	**Stage 1**	**Stage 2**	**Stage 3**	**p value**
Radiation modality ^∞^	IMRT 4/4	IMRT 3/3	IMRT 1/5	p = 0.03*
			Conventional 4/5	
Radiation dose (Gy), mean ± SD ^§^	52.5 ± 5	60 ± 0	59 ± 9.86	p = 0.312^¶^
Average time from radiation to diagnosis in months (range)	6.47 (3.0–12.0)	9.83 (6.0–11.9)	52.35 (9.0–129.6)	p = 0.056^¶^

^∞^ Modality information is missing on 1 patient with Grade 1 and 1 patient with Grade 3 disease.

^§^ Dose information missing in 2 patients with Grade 3 and 1 patient with Grade 1 disease.

* Freeman-Halton extension of the Fisher exact probability test.

^¶^ One-way Analysis of Variance (ANOVA).

*IMRT* intensity modulated radiation therapy.

*SD* standard deviation.

Adjuvant concomitant chemoradiotherapy was administered in 3 patients and resulted in stage 2 or 3 diseases.

All patients had appropriate institutional dental screening, but only in one patient dental procedures were performed following radiation, and could be implicated as a culprit of ORN.

### Treatment: HBO and surgery

HBO was used in 57.1% of patients as an adjunct to surgical debridement. It was used for all stages of disease (Table 4). In a number of cases, however, this treatment was not sufficient to control the disease.

**Table 4:** Hyperbaric oxygen therapy use by stage

**Stage**	**Number of patients receiving HBO therapy from total number of patients with that stage**
1	3/5 (60%)
2	1/3 (33.3%)
3	4/6 (66.7%)
Total	8/14 (57.1%)

*HBO* hyperbarix oxygen, *N*/*A* not available.

Antibiotics were almost always employed in the management of stage 2 and 3 ORN. If there was a delay leading to definitive surgery, long-term antibiotics were used. They were stopped after clinical and radiographic ORN resolution or complete healing after flap reconstruction. The choice of antibiotics varied, but usually included Gram-negative coverage for oral pathogens. Most commonly used antibiotics were (dose for a 70 kg adult patient): Amoxicillin 500 mg three times per day, Clindamycin 300 mg four times per day, or Keflex 500 mg four times per day.

Patients, whose disease was not amenable to control via conservative measures, underwent surgical procedures (Table 5). Most of patients with stage 1 disease underwent local debridement procedures to the margin of the healthy tissue. Patients with stage 2 disease had segmental resections or free tissue transfer reconstructions, whereas patients with stage 3 disease underwent more extensive mandibulectomies and free tissue transfers.

**Table 5:** Surgical procedures for primary tumor and ORN management

**Stage**	**Tumor**	**Primary ORN surgery**	**Other ORN surgery**
1	T3N0 soft palate	N/A	N/A
	T2N0 retromolar trigone	N/A	N/A
	T4N2 tonsil	Local debridement	N/A
	T2N0 retromolar trigone	Local debridement	N/A
	T3N0 tongue	Marginal resection	Local debridement, dental procedures
2	T3N0 tongue	Mandibular resection, sequestrectomy	Sequestrectomy
	T2N1 tongue	Segmental resection, FFF	Iliac crest bone graft
	T4N2 tonsil	Segmental resection, osteocutaneous lat dorsi and parascapular free flap	N/A
3	T2N2 tongue	Sequestrectomy and saucerisation	Mandibular resection; Re-resection, FFF, pectoralis major flap
	T2N2 parotid	Partial mandibulectomy, FFF	RFFF
	T4N1 tonsil	Mandibulectomy, FFF, pectoralis major flap	Iliac crest bone graft
	T2N0 tonsil	Mandibular resection, reconstructive plate	N/A
	T2N0 tonsil	Segmental resection, reconstructive plate	Replacement plate, iliac crest bone graft
	T2N0 buccal mucosa	Marginal resection	Local debridement

*ALT* anterolateral thigh free flap, *FFF* fibula osteocutaneous free flap, *N*/*A* not available, *ORN* osteoradionecrosis, *RFFF* radial forearm free flap.

The complexity of surgical care can be summarized by the use of free tissue transfers per patient with the specific stage of ORN (Table 6). None of the patients with stage 1 disease required free tissue transfers. There were a total of 2 free flap procedures in 3 patients with stage 2 disease. On the contrary, there were 6 free tissue transfers in 6 patients with stage 3 disease, including 2 cases, where two simultaneous free flaps were utilized during a single procedure in either primary or secondary ORN reconstruction. One flap was required for external skin cover due to markedly contracted soft tissue envelope. The second flap was provided with either a pectoralis major myocutaneous flap or additional fasciocutaneous flap based on recipient vessel availability. There were no flap failures encountered in this series.

**Table 6:** Reconstructive surgical techniques by type

**Stage**	**Total flaps (two simultaneous flaps)**	**2nd free flap (two simultaneous flaps)**	**Total # flaps per total # patients per stage**
1	0	0	0/5
2	2	0	2/3
3	3 (1)	3 (1)	6/6

Follow up was available for 13 of 14 patients for an average of over 2 years. There was no evidence of recurrent or residual cancer in any patient. No clinical evidence of ORN was present in 84% of patients.

## Discussion

We present our experience with treating patients with ORN at two departments at a large urban academic center in the period of 6 years.

Despite close to a century has passed since the first description of ORN shortly after the discovery of x-rays, there is a multitude of controversies on the subject in the literature, including about its definition and best management options [1].

Historically, several factors can influence ORN development, including the site and size of the tumour, radiation dose, type of mandibular resection, injury, or dental extractions, infection, immune deficiencies, and malnutrition. It is necessary to keep in mind that many patients with oral cancer have other serious diseases and have often had a long history of alcohol and tobacco misuse, which, in combination with malnutrition and poor oral hygiene, place these patients at higher risk of developing ORN. In our series, 13 of 14 patients had primary tumors in the oral cavity. There was no significant correlation of ORN stage with tumor site, or T, N, or AJCC stage.

Radiation is the main etiologic factor in the development of ORN, in a dose-dependent manner. The incidence has decreased over years, from an estimated 15% in 1970s to 0-5% in 2000s. In our series, the incidence rate of ORN was 0.84%.

ORN is rare with conventional radiation doses of less than 60Gy, while the rate in those irradiated with >60 Gy is around 12% [8] IMRT can reduce the dose delivered to the salivary glands and reduce the rate of xerostomia, as well as other radiation related toxicities. A study from the University of Michigan reported no cases of ORN of the mandible at a median follow-up of 34 months after IMRT for head and neck cancer, using a strict prophylactic dental care policy [9]. A recent study from the Memorial Sloan-Kettering Cancer Center showed that ORN is rare with the use of IMRT [10]. Similar results were found in another European study [11]. In a review of 18-year literature evaluating the impact of cancer therapies on prevalence of ORN, Peterson et al. reported a weighted ORN prevalence of 7.4% for conventional radiotherapy, 5.1% for IMRT, 6.8% for chemoradiotherapy and 5.3% for brachytherapy [12].

It can be extrapolated that IMRT would also result in less severe cases of ORN, when it does develop. In this study, IMRT was associated with less severe ORN stage compared to conventional radiotherapy. There was no correlation identified between radiation dose and severity of ORN, presumably due to the small number of patients in the study.

Treatment of ORN is a challenging problem. There are a variety of treatment options, and no universal approach to management, as this depends on institutional or individual experiences. At our institution, Head and Neck and Oral Maxillofacial Surgeons together manage patients with ORN.

A treatment that has been in the center of much controversy is HBO. In 1985, Marx developed both a classification scheme and a treatment protocol for ORN based on the number and sequence of HBO dives [3,13]. This protocol has been used with variable success in many studies. An analysis of evidence of the management of ORN of the mandible demonstrated that HBO continues to be used as an adjunct in the management of ORN, while its clinical usefulness remains controversial [6]. HBO alone has minimal if any benefit in the treatment of advanced ORN [7].

An updated Cochrane review on HBO for the treatment of the late effects of radiotherapy found some evidence that HBO therapy is more likely to achieve mucosal coverage with ORN (RR 1.3; 95% CI 1.1 to 1.6, P = 0.003) [14]. Single studies included in this review demonstrated a significantly increased chance of improvement or cure for HBO therapy following both surgical flaps and hemi-mandibulectomy, while there was also a significantly improved probability of healing irradiated tooth sockets following dental extraction.

In our series, HBO was employed along with surgical debridement in the management of 8 of 14 patients, with all stages of disease. Ultimately, in the majority of cases, HBO in combination with debridement did not prevent the progression of disease in stage 2 and 3 patients, who required extensive surgical procedures to control the disease.

Management of stage 2 disease remains of most significant interest. Stage 2 is defined as exposed alveolar bone that does not respond to HBO, and requires sequestrectomy or saucerization. There were 3 patients in stage 2 ORN in our series. All of them were treated with adjuvant IMRT at 60 Gy. In this series, decisions to treat by either sequestrectomy or free tissue transfer were dictated by disease extent. A patient with T3NO oral tongue cancer had two areas of exposed alveolar bone, which did not bleed with manipulation. He was treated with marginal mandibular resection and sequestrectomy, followed by postoperative HBO, to which he responded. The second patient with T2N1 tongue cancer had significant exposed alveolar bone without pathologic fracture. He was in significant pain and had pronounced facial edema. A decision was made to perform a segmental mandibular resection and fibular osteocutaneous free flap reconstruction, as it was feared that this patient would not respond to local procedures. Finally, the third patient with stage 2 disease had T4N2 tonsillar squamous cell carcinoma. He had an infected non-union and persistent oro-cutaneous fistula. Given the amount of involvement, segmental resection of the mandible and reconstruction with a mandibular place and osteocutaneous latissimus dorsi and parascapular free flaps were undertaken.

Jacobson et al. have recently summarized the paradigm shifts in the ORN management [7]. Importantly, they have emphasized the conservative approach to management of stage 1 disease, while treatment with antibiotics, transoral debridement or sequestrectomy, and HBO therapy was reserved for stage 2 disease. Patients with stage 3 disease are treated aggressively by surgical resection of all diseased hard and soft tissue and immediate reconstruction with free tissue transfer.

**Figure 1:**
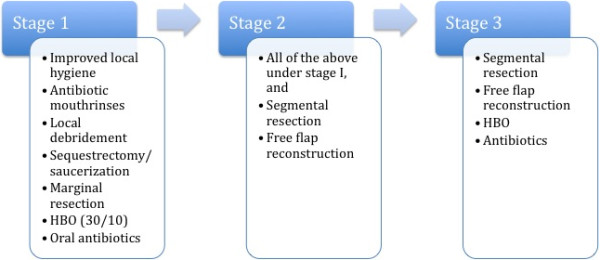
An outline of the treatment algorithm by stage of osteoradionecrosis (ORN).

Surgical treatment of patients in our series closely followed these principles. Our approach to ORN treatment is summarized in Figure 1. The majority of patients with stage 1 ORN were treated conservatively and with local debridement. Surgical care escalated to sequestrectomy and marginal resection with reconstruction in stage 2 patients. Most patients with stage 3 disease underwent radical excision of ORN with free tissue reconstruction. Of note, several patients with stage 3 disease required a second flap, which in 2 cases was a simultaneously performed two free flap procedures. Expectedly, the number of free flaps used increased corresponding to disease stage.

## Conclusions

We advocate an aggressive approach to the treatment of ORN with liberal use of resection of diseased bone and reconstruction with vascularized free tissue transfer. In our experience and in agreement with the recent literature, HBO alone was not sufficient to control ORN progression. In a few of the most advanced cases, two simultaneous flaps were required to provide a vascularized bed after radical resection of all diseased tissues, and to provide soft tissue coverage and reduce tension across suture lines to avoid venous congestion in the microvasculature.

## Abbreviations

HBO: Hyperbaric oxygen; IMRT: Intensity-modulated radiotherapy; ORN: Osteoradionecrosis.

## Competing interests

The author(s) declare that they have no competing interests.

## Authors’ contributions

AG summarized and analyzed the database, drafted the manuscript. KW collected and summarized the database. IP participated in the coordination of the study, provided important information about irradiated patients. NB participated in the design of the study, collected and summarized part of the database. DE participated in the design of the study. KH conceived of the study, participated in its design and coordination and helped to draft the manuscript. All authors read and approved the final manuscript.

## References

[B1] Epstein JB Wong FL Stevenson-Moore P Osteoradionecrosis: clinical experience and a proposal for classification YJOMS 1987 45 104 110 10.1016/0278-2391(87)90399-5 3468211

[B2] Teng MS Futran ND Osteoradionecrosis of the mandible Curr Opin Otolaryngol Head Neck Surg 2005 13 217 10.1097/01.moo.0000170527.59017.ff 16012245

[B3] Marx RE Osteoradionecrosis: a new concept of its pathophysiology YJOMS 1983 41 283 288 10.1016/0278-2391(83)90294-x 6572704

[B4] Delanian S Lefaix J-L The radiation-induced fibroatrophic process: therapeutic perspective via the antioxidant pathway Radiother Oncol 2004 73 119 131 10.1016/j.radonc.2004.08.021 15542158

[B5] Annane D Hyperbaric oxygen therapy for radionecrosis of the jaw: a randomized, placebo-controlled, double-blind trial from the ORN96 study group J Clin Oncol 2004 22 4893 4900 10.1200/JCO.2004.09.006 15520052

[B6] Pitak-Arnnop P Sader R Dhanuthai K Masaratana P Bertolus C Chaine A Bertrand JC Hemprich A Management of osteoradionecrosis of the jaws: an analysis of evidence Eur J Surg Oncol 2008 34 1123 1134 10.1016/j.ejso.2008.03.014 18455907

[B7] Jacobson AS Buchbinder D Hu K Urken ML Paradigm shifts in the management of osteoradionecrosis of the mandible Oral Oncol 2010 46 795 801 10.1016/j.oraloncology.2010.08.007 20843728

[B8] Nabil S Samman N Incidence and prevention of osteoradionecrosis after dental extraction in irradiated patients: a systematic review Int J Oral Maxillofac Surg 2011 40 229 243 10.1016/j.ijom.2010.10.005 21115324

[B9] Ben-David MA Diamante M Radawski JD Vineberg KA Stroup C Murdoch-Kinch C-A Zwetchkenbaum SR Eisbruch A Lack of osteoradionecrosis of the mandible after intensity-modulated radiotherapy for head and neck cancer: likely contributions of both dental care and improved dose distributions International Journal of Radiation Oncology*Biology*Physics 2007 68 396 402 10.1016/j.ijrobp.2006.11.059 PMC2702207 17321069

[B10] Gomez DR Estilo CL Wolden SL Zelefsky MJ Kraus DH Wong RJ Shaha AR Shah JP Mechalakos JG Lee NY Correlation of osteoradionecrosis and dental events with dosimetric parameters in intensity-modulated radiation therapy for head-and-neck cancer Int J Radiat Oncol Biol Phys 2011 81 207 213 10.1016/j.ijrobp.2011.02.003 21570202

[B11] Studer G Studer SP Zwahlen RA Huguenin P Grätz KW Lütolf UM Glanzmann C Osteoradionecrosis of the Mandible Strahlenther Onkol 2006 182 283 288 10.1007/s00066-006-1477-0 16673062

[B12] Peterson DE Doerr W Hovan A Pinto A Saunders D Elting LS Spijkervet FKL Brennan MT Osteoradionecrosis in cancer patients: the evidence base for treatment-dependent frequency, current management strategies, and future studies Support Care Cancer 2010 18 1089 1098 10.1007/s00520-010-0898-6 20526784

[B13] Marx RE Johnson RP Kline SN Prevention of osteoradionecrosis: a randomized prospective clinical trial of hyperbaric oxygen versus penicillin J Am Dent Assoc 1985 111 49 54 3897335 10.14219/jada.archive.1985.0074

[B14] Bennett MH Feldmeier J Hampson N Smee R Milross C Hyperbaric oxygen therapy for late radiation tissue injury Cochrane Database Syst Rev 2012 5 005005 10.1002/14651858.CD005005.pub2 16034961

